# Announcements of Death During the COVID‐19 Pandemic: A Qualitative Study of Family Experiences

**DOI:** 10.1111/hex.70221

**Published:** 2025-03-12

**Authors:** Axelle Avazeri, Juliette Bonin, Axelle Maneval, Philippe Vorilhon

**Affiliations:** ^1^ Department of General Practice Université Clermont Auvergne, UFR Medicine Clermont‐Ferrand France; ^2^ Université Clermont Auvergne UR ACCePPT Clermont‐Ferrand France; ^3^ Billom Hospital Center Billom France; ^4^ Unit of palliative care, Centre Hospitalier Universitaire (CHU) Clermont‐Ferrand nUiversité Clermont Auvergne Clermont‐Ferrand France; ^5^ Research and Innovation Department Centre Hospitalier Universitaire (CHU) Clermont‐Ferrand Clermont‐Ferrand France

**Keywords:** communication, COVID‐19, end‐of‐life, interpretative phenomenology, qualitative research, semistructured interview

## Abstract

**Introduction:**

Health restrictions resulting from COVID‐19 made it more difficult for families to mourn. The death announcement is a significant moment for families. The aim of this study was to explore the experiences, perceptions and expectations of families who were informed of the death of a close relative in the hospital, at home or in a care home for dependent elderly people (EHPAD) during the COVID‐19 pandemic.

**Methods:**

This qualitative study was based on interpretative phenomenology and involved individual semidirected interviews with families of patients who died in 2020 in the Auvergne region, in hospitals, at home and in establishments for dependent elderly people, with double analysis.

**Results:**

Three themes emerged from the 17 interviews. Families' experiences of the announcement of the death of a loved one were complex due to the pandemic as well as the corresponding health restrictions and new forms of communication, thus leading to fear, helplessness, frustration, sadness, anger, guilt and loneliness. High‐quality communication with caregivers, both at the announcement of death and beforehand, was essential. The families highlighted various areas for improvement: family visits with the patient, effective telecommunication, close emotional support after the announcement, reflection by caregivers on their own defence mechanisms in response to death, and a care system that revalues *‘taking care’*.

**Conclusions:**

Families' experiences with receiving news regarding the death of a close relative during the pandemic depended on the quality of their communication with caregivers, which could mitigate the traumatic impacts of health measures and relational deprivation. Adjustments are necessary in the event of similar pandemic situations.

**Patient or Public Contribution:**

The conception of our study was based on discussions with families in hospitals or general practice who reported their difficulties and suffering at the time of the death of a close relative during the COVID‐19 pandemic. We reasoned that understanding the experiences of people who had experienced the death of a loved one in this health context would enable us to determine whether relationships with caregivers met their expectations or whether they needed to be adapted. To place the experience of families at the centre of this research, a participatory research method with a qualitative approach was used. The families of patients who died in 2020 in the Auvergne region during the COVID‐19 pandemic were included in the study. A list of families has been established from the healthcare software of a hospital, a home for dependent elderly people and two multiprofessional primary care practices. Twenty‐six relatives of patients were contacted, and 17 interviews were conducted. Seven people refused to participate, one person did not participate in the follow‐up, and one person could not be contacted.

Based on discussions with relatives of deceased patients and a literature search, we established an interview guide. The families approached volunteered to share their experiences and subsequently validated the study data.

## 1 Introduction

The conditions under which the news of a death is announced remain permanently in the memories of the bereaved [1, 2].

In January 2020, the World Health Organization (WHO) declared an international public health emergency due to the growing number of countries where the COVID‐19 epidemic was spreading [3]. Health restrictions and social distancing aimed at limiting the spread of the virus to isolated patients at the end of their lives, both in hospitals and at home [4, 5, 6, 7, 8, 9]. Although the care context was different, there were similarities in the situations experienced by families at the moment of the death of a loved one (protective equipment for independent caregivers, limited home visits, teleconsultations, reduced physical contact, restrictions on family travel and isolation of the caregiver).

In this context, families experienced a lack of kindness towards the deceased and believed that the farewell process was rushed or even nonexistent [10]. They also experienced a lack of exchange with caregivers, which represents a source of distress that affects their grieving process [11, 12].

Guides and training sessions involving role‐playing have previously been developed to promote the communication skills needed to communicate bad news in medical contexts [1, 13]. However, COVID‐19 has led to the emergence of new obstacles to effective communication: widespread fear and uncertainty, increasing work demands for caregivers, and the need to employ unfamiliar forms of telecommunication [14].

A better understanding of families' experiences with the announcement of death during the pandemic would make it possible to limit such painful experiences and to develop novel recommendations with a focus on families. Studies on this subject, particularly from a qualitative perspective, have been limited [15, 16].

The aim of this study was to explore the experiences, perceptions and expectations of families who were informed of the death of a close relative in a hospital, at home or in residential establishments for dependent elderly people (EHPAD) during the COVID‐19 pandemic.

## 2 Methods

### 2.1 Type of Study

Qualitative research based on interpretive phenomenology was used to explore families' experiences of being informed of the death of a loved one during the COVID‐19 pandemic. Semistructured individual interviews with families were conducted because of the intimate nature of the subject under investigation. These interviews offered the necessary space to listen to participants' life stories while addressing the various desired themes. The study was reported using the *COnsolidated criteria for REporting Qualitative research* (COREQ) grid (Supporting Information S1) [17]. The positionality of the research team is presented in Supporting information S2.

### 2.2 Study Population

A purposive sampling approach that was targeted and homogenous in terms of the experience of the phenomenon under study was employed to investigate the families of patients who died in 2020 in the Auvergne region. We chose these 3 places of death, hospital, EHPAD and home, to have criteria for the diversity of families interviewed with similar experiences. This research relied on the health software employed by Issoire Hospital, two EHPAD facilities (Issoire and Saint Pourçain‐Sur‐Sioule) and 14 general practitioners working in two multiprofessional medical centres (Gannat and Saint Pourçain‐Sur‐Sioule). A list of 26 families was obtained. For the constitution of a purposive sample, descriptive information was collected from these families, including gender, age (≥ 18 years), occupation, place of residence (urban, semirural or rural), relationship with the deceased, and circumstances of death (such as the age of the deceased, whether the death was sudden or expected, whether the death was linked to COVID‐19, and whether the death occurred at home, in an EHPAD or in hospital). To limit the possibility of oversensitive reactions, we fixed a 6‐month delay between death and the interview.

The families were contacted by telephone; during this conversation, they were given a presentation of the study and explanations for their participation in interviews and, at the 2nd phase, in the review of the study data. If they agreed to participate, an appointment for the interview was then made either at their home or by phone according to their wishes. A letter of information explaining the aims and progress of the study was sent to them by mail.

### 2.3 Data Collection

The interview guide was developed in accordance with the recommendations of Blanchet et al. [18] A literature search was subsequently conducted to investigate the subject under study. The questions addressed aspects such as the circumstances of the death and its announcement, feelings, helping factors and difficulties associated with this situation, the expectations of the caregivers and prospects for improvement when the death was announced (Table 1). The questionnaire evolved over the course of the study in response to misunderstandings and was used flexibly to allow families to express their experiences. It was tested with a relative who was not included in the study and adjusted at the time of the fourth interview. In light of some misunderstandings and the fact that some of the answers diverged from the initial subject, some questions were reformulated, reminders were used, and a question on the needs of the families was added (see Supporting Information S3).

**Table 1:** Initial interview guide.

Initial interview guide
1. **Sociodemographic data of the participants' relatives:** gender, age, profession, place of residence, relationship with the deceased person, and support person.
2. **Tell me the circumstances of your loved one's death:** gender, age, date, circumstances of death,and place (hospital, home, nursing home)
3. **How did you learn of the death of your loved one and tell me about this announcement?**
4. **How did you experience this announcement of the death?**
5. **During this announcement, what did you find positive or negative?**
6. **What do you think could have improved during this announcement?**
7. **Do you see any other things you would like to discuss about this?**

The interviews were conducted by J.A., supervised by author J.B., audio‐recorded and then transcribed manually, in full and anonymously using Word software. J.A. had never met any of the participants before. Nonverbal language was noted.

### 2.4 Data Analysis

The transcripts of the various interviews were analysed using a phenomenological interpretative analysis approach (IPA) [19, 20]. This approach focused on the participants' experiences and was applied independently to each interview. Various themes emerged, which were then grouped into superordinate themes; these themes were articulated coherently in light of the research objective. These coding phases were carried out individually by each of the two researchers (J.A. and J.B.) before the data were pooled to respect the triangulation of the data. The main data were communicated to the participants to verify the degree of consistency between the research and their representations. An explanatory schema was produced following the analysis, articulating the superordinate themes in a coherent way and attempting to reflect the meaning that the participants gave to their lived experience.

### 2.5 Ethical and Regulatory Aspects

This study did not fall within the scope of French regulations governing research involving human subjects (RIPH). A favourable ethical opinion was obtained from the Comité de protection des personnes (CPP) Sud Est VI. The participants signed a written consent form. If the investigator detected pathological or complicated bereavement during the interview, she suggested that the participants meet with a clinical psychologist (A.M.) used to meet families in the palliative care department. This psychologist was not requested by any of the participants.

## 3 Results

Twenty‐six relatives of patients who died in 2020 were contacted. Seventeen interviews were conducted between September 2021 and March 2022. Seven people refused to participate, one person did not participate in the follow‐up, and one person could not be contacted. The characteristics of the participants are presented in Table 2. The interviews lasted an average of 40 min each. Data saturation was reached after 14 interviews, and 3 additional interviews were carried out to verify this fact. The relevance of the data was validated by all the participants, and new data were added at this time. In total, three themes emerged from the analysis of the interviews.

**Table 2:** Characteristics of the participants.

Participants	Gender	Age (years)	Profession	Living place	Link with the deceased	Circumstances of death				Interview procedure
						Age of the deceased (years)	Death expected or sudden	Place of death	COVID‐19 ‐related death	
P1	H	74	Retired (Metalworker)	Rural	Brother	86	Expected	Hospital (medicine department)	No	Home
P2	H	83	Retired (Worker)	Semirural	Friend	98	Sudden	EHPAD	No	Home
P3	H	62	Retired (Banker)	Semirural	Son	90	Expected	Hospital (medicine department)	Yes	Home
P4	F	58	Commercial	Urban	Daughter	87	Expected	EHPAD	No	Phone
P5	F	71	Retired (Farmer)	Rural	Daughter	97	Expected	Hospital (medicine department)	No	Home
P6	F	63	Retired (Secretary)	Semirural	Wife	64	Sudden l	Home	No	Home
P7	F	70	Retired (Specialist educator)	Rural	Wife	69	Expected	Home	No	Home
P8	F	78	Retired (Teacher)	Urban	Wife	83	Sudden	Hospital (intensive care unit)	Yes	Phone
P9	F	70	Retired (Care assistant)	Semirural	Daughter	94	Expected	EHPAD	No	Home
P10	H	65	Retired (Engraver)	Semirural	Son	95	Sudden	EHPAD	No	Home
P11	F	71	Retired (Secretary)	Rural	Wife	73	Sudden	Hospital (medicine department)	No	Phone
P12	F	73	Retired (Retailer)	Semirural	Wife	75	Expected	Hospital (medicine department)	No	Phone
P13	H	60	Doctor	Semirural	Son	93	Expected	Hospital (medicine department)	No	Phone
P14	H	60	Farmer	Rural	Son	87	Expected	Home	No	Home
P15	F	58	Teacher	Urban	Daughter	82	Sudden	Hospital (intensive care unit)	Yes	Phone
P16	F	68	Retired (Funeral director)	Semirural	Wife	66	Sudden	Hospital (emergency department)	No	Phone
P17	F	65	Retired (Saleswoman)	Semirural	Daughter	91	Brutal	EHPAD	Yes	Phone

Abbreviations: EHPAD, établissement d'hébergement pour personnes âgées dépendantes (accommodation for dependent elderly people); SPC, socioprofessional category.

### 3.1 An Upheaval in Participants' Experiences With the Announcement of Death

As expected, the experience of the announcement of death was linked to the circumstances of death and the life history of the families.

It is only once in your life that you're told your mother has died […] It's like a tsunami.

(P4)

For families, the period under investigation was described as unprecedented and was associated with many uncertainties. The climate during this period was characterised by anxiety, which was intensified by the media. The people surrounding the patient feared that they could become vectors for the virus. COVID‐19 was highly prioritised, to the detriment of other diseases. This situation made it more difficult for families to cope with the news of the patient's death.

All that mattered was COVID […] People with cancer, we do not give a damn about them now.

(P6)

The ban on visits to the hospital and EHPAD caused incomprehension among patients and led to depressive syndromes. Families expressed helplessness in the face of their loved one's isolation. This situation caused the families' experiences to become even more complicated when the death was announced. Sadness, anger, guilt and regret at the time of the announcement were amplified.

When they were isolated in the rooms, that was the last straw! […] She was abandoned […] She did not understand: “We're plague victims” […] I say to myself, what did she think of us last?

(P10)

While choosing to die at home could increase the comfort of dying people who are surrounded by their loved ones, the patient's family feels lonely and unsupported. They regretted delays in the provision of home help, dysfunctional coordination with the hospital, excessive workloads faced by independent caregivers, and limitations in terms of contact with caregivers due to precautions related to COVID‐19. Families reported that they found it difficult to serve as replacements for caregivers, a situation that led to physical and mental exhaustion.

No one gave any sign of life […] You have to have guts […] She had just died, so I gave her her dentures.

(P14)

The families mentioned their frustration with the process of communicating with caregivers, which was complicated by the need for protective equipment and facilitated by telecommunication, thus highlighting the importance of physical contact with patients. For these families, communicating with a doctor via telecommunication led to mistrust. Some families even described feelings of abandonment by the doctor.

We only spoke on WhatsApp. It is not the same thing […] He said: “No, I do not want this anymore, I cannot touch them, I cannot talk to them, I do not want this anymore, […] You talk to him by videoconference, but you do not feel anything…”

(P6)

Health restrictions interfered with the attention given to the deceased, thus complicating the experience of death. Immediate burial was perceived as brutal and depersonalising. The impossibility of religious rites, out‐of‐touch funeral directors, delayed funerals, restrictions on the number of people at the funeral and measures aimed at distancing people during the funeral were all harshly criticised.

Given the number of deaths that occurred during this period, the families described a new relationship with death. They noted that death was mentioned on a daily basis in conversations, in the media and on social networks, such as through announcements regarding the number of deaths or the difficulties involved in organising funerals. One participant even discussed ‘breaking a taboo’.

We never saw her again. She was in a cover, the coffin was closed, and that was that.

(P17)

They tell you very clearly that far too many people died at the same time and that the crematoria were saturated.

(P4)

### 3.2 Communication With Caregivers at the Heart of the Bereavement Process

Families emphasised that good communication with caregivers improved their experience of the news of the death of a loved one during the pandemic. However, some participants felt that communication was poor and abrupt, thus exacerbating the stupefaction, sadness, loneliness and even anger they experienced at the time of the announcement.

If I have one thing to ask of hospitals and nursing homes, it is good communication. Whether it is good news or bad, whether it is peaceful […] It is truly important, when you're facing bereavement, in any case.

(P15)

In the evening, she was watching TV. In addition, then they phoned me. They said: “She's passed away”. That was it.

(P2)

The establishment of a relationship with the person announcing the death was comforting for the family. Relatives valued communication skills on the part of the person announcing the death; such skills included the ability to inform families of the death in a tactful way, the acknowledgement of emotions, the capacity to ease guilt, the willingness to discuss the deceased loved one and his or her final moments, and availability. Guidance regarding administrative procedures was also appreciated.

The bond of trust and the quality of the communication seemed to be prioritised over the status of the person announcing the death (doctor, resident, or nurse) and the means of communication (face‐to‐face or telephone).

Families also highlighted their need to be warned of the expected death and to be informed about the moments preceding the patient's death.

She [the doctor] was beside me, she took me by the shoulder […] No, frankly, I did not get the feeling that she did not care, that my husband was just another patient.

(P6)

No, no one said to me: “He's at the end of his life”. No, not at all, I have never heard that word. Maybe I would have prepared myself better.

(P11)

### 3.3 Prospects for Improvement

The families' expectations were met with resignation. They explained this situation with respect to the powerlessness of caregivers in response to death and the need for health measures to alleviate the urgency of the situation as a result of COVID‐19.

They affirmed their need to be present with the patient at the end of life and to play a supportive role in that context. Being at the patient's side made it easier for them to understand the situation and to say farewell. While telecommunication was not a substitute for physical contact, the development of effective videoconferencing between families and patients in hospitals and nursing homes was mentioned, particularly in situations in which visits were restricted and families were far from the dying person.

It would be good if there was someone on the wards who was not necessarily a caregiver in end‐of‐life situations such as this, who could contact the families by video when the patients could not do it.

(P15)

The families felt that the caregivers distanced themselves from the families, thereby employing modesty and defence mechanisms in response to death; this situation had a negative impact on their communication. They were aware that caring for a dying patient and his family represented a source of suffering for the caregivers. They suggested reconsidering their attitudes towards death with the goal of improving their communication. A call or message from the patient's general practitioner (GP) following death was appreciated.

Doctors do not get used to death, but well… they have to do it, that is life, that is the way it is […] The doctor could have talked to us the day she died for a little while.

(P1)

The inability of the family to be at the patient's side was associated with an emphasis on ‘taking care*’*. Families felt that caregivers were overwhelmed by the pandemic. They highlighted the intrusion of economics into medicine, particularly with respect to nursing shortages and attempts on the part of healthcare systems to ensure profitability.

They were overloaded; I'm not blaming them, but it was atrocious […] It is not even a question of humanity; it is inadequate budgets!

(P15)

People who were close to the deceased described feeling upset or even traumatised when they received news of death, and the corresponding loneliness was exacerbated by confinement. The families felt the need to talk and share with others. They turned to relatives, GPs, psychologists, relaxation therapists, associations and other bereaved people.

After going through that, I would have needed to talk, to get it all out, because at the time, we did not have enough… [listening] even if there was a bit.

(P15)

## 4 Discussion

### 4.1 Main Results

**Figure 1:**
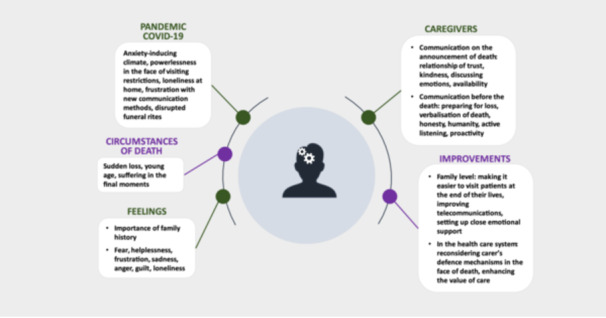
Explanatory schema: family experiences and expectations.

Although this issue was topical during the COVID‐19 pandemic, the perspectives of families on announcements of the death of a loved one have received little attention. Their experiences were complicated by the pandemic as well as their health restrictions and novel ways of communicating, which led to fear, powerlessness, frustration, sadness, anger, guilt and loneliness. Against this backdrop, the families would have liked to have engaged in high‐quality communication with caregivers, both when the death was announced and beforehand, which could help them feel more accepted. The following areas for improvement were identified: the presence of families at the patient's side at the end of life, the use of effective telecommunication, the provision of close emotional support after the announcement, reflection by caregivers on their own defence mechanisms in response to death, and a focus on *‘*caring’ in the context of care provision (see Figure 1 for an explanatory model).

### 4.2 Strengths and Limitations

Our study has several limitations. First, this research was conducted solely in the Auvergne region. Although the organisation of hospitals, nursing homes and general practitioners in this region was in line with the national health measures implemented at this time, the first wave of COVID‐19 nevertheless affected the regions of France in different ways.

Among the 26 family representatives approached, 17 agreed to participate in this research. Among the 7 potential participants who refused, 4 indicated that they found it difficult to consider their painful memories. In addition, all deaths involved elderly patients. As a result, some experiences may not have been reported. However, despite these refusals, the criteria related to diversity were satisfied.

The interviewer introduced herself as a medical resident, which may have led participants to adopt a certain restraint or to influence their comments on social desirability. However, this approach could also have anchored the relationship between these two parties in the trust that is necessary to express emotions. Memory bias may have impacted the results of this study to some degree, since families were asked to recall the death of their relative, which had occurred 2 years earlier.

Nine out of the 17 interviews were conducted by telephone, which was the most suitable method for complying with barriers pertaining to participants' choices or geographical distance. Nonverbal elements may thus have been lost; however, the anonymity offered by this approach could facilitate free expression on the part of the participants. The intonation and emotions perceived by the researcher were accurately transcribed.

The qualitative IPA method used to conduct this research, which involved individual interviews, enabled us to gain a better understanding of the families' intimate experiences. The validity of the study was strengthened by several factors. Coding was performed by two researchers, thus limiting the impacts of personal interpretations. The participants validated the data without any disagreements or new information. External validity was enhanced by carefully selecting a varied panel of participants.

### 4.3 Comparison With Data Reported in the Literature

The participants all cited the COVID‐19 pandemic as a factor influencing their experience of the announcement, in addition to the circumstances of death and life history, which are factors well described in the literature [1].

Restrictions on visits to the hospital and EHPAD represented a source of despair for families, and this finding was consistent with the results of several previous studies [11, 15, 21, 22]. The risks of anxiety and depressive syndrome are increased; in addition, these risks are associated with negative perceptions of the quality of care and communication with caregivers [11]. The main difficulty reported by the families of patients who died at home pertained to exhaustion resulting from the absence of the usual continuity of care at home, as revealed by an observational study [6].

The perceptions of families regarding videoconferencing with the patient were mixed, as described in another qualitative study; these perceptions involved disappointment at the lack of access to such technology, comfort due to seeing their loved one, and recognition of the help provided by caregivers as well as frustration owing to the lack of physical contact, suffering in response to upsetting images, etc. [23]. The families included in our study were more distrustful of telecommunication with caregivers and felt abandoned. These feelings have been reported in other studies: the physical distance and imperfections of the technology represent a hindrance during certain stages, such as those involving empathy and the recognition of emotions, since this technology is limited to technical information [15, 24]. Protective equipment such as masks also negatively affects communication and the ability to convey emotions such as empathy [25]. The families who were investigated regretted the fact that certain funeral rituals could not be performed, although such rituals have been recognised as being helpful regarding mitigating grief, irrespective of whether they feature a religious dimension [26].

Against this backdrop of fear, powerlessness, frustration, sadness, anger, guilt and loneliness, families indicated that the pandemic had made their experiences of learning about the death of a loved one more complex. Some of the feelings described by the participants were common feelings of grief [1]. While qualitative research cannot establish a causal link, our study does suggest that the unprecedented period of the COVID‐19 pandemic and its restrictions were factors in the severity of families' grief reactions to the news of the death of a loved one. Indeed, the pandemic has affected bereaved people in several ways other than causing a loved one's death. It presented itself as a major traumatic event, putting others' and one's own life in danger and bringing about multiple types of losses at the same time, including losses in human lives, property, income, and overall wealth [27]. Studies have revealed that psychological adjustment and acute grief reactions became more complicated during the pandemic, regardless of whether the deaths in question were related to COVID‐19 [28, 29]. A systematic review indicated that the probability of prolonged bereavement was much greater in the case of nonnatural deaths, particularly among adults who were bereaved due to COVID‐19 [30, 31].

In response to this unprecedented situation, some of the families included in our study highlighted the need to share their experiences, thereby overlooking taboos pertaining to death. This finding was in line with the idea of sharing with the social environment as a means of protecting the bereaved, which could enable them to move on from their grief [32, 33]. Although the loss of a loved one is a private and intimate matter, it seems to take on a new collective dimension in this context [34]. The role of caregivers in the bereavement process was highlighted in our study. That is, good communication made it easier for the bereaved to cope with the news of death, which was already known, in any situation, without pandemic restrictions [1]. In his study, Jurkovich claimed that for families, a caring attitude on the part of the person announcing the death was the most important aspect of communication [35]. In another qualitative study, families also reported that caregivers did not engage in sufficient communication regarding death, and they highlighted the importance of preparing for the loss, requiring a cognitive, affective and behavioural dimension [36]. However, participants identified communication with caregivers during the announcement of death and upstream as a major contextual factor during the COVID‐19 pandemic, which has been suggested in other studies [11, 22, 33, 37]. Proactive, empathetic, transparent and regular communication by caregivers during visitation restrictions and social distancing policies allowed families to feel included in care decisions and alleviated their emotional distress [22, 38]. The families included in this study encouraged caregivers to identify their own difficulties with the goal of helping them cope with the patient's death more effectively, an approach that is in line with a French recommendation regarding attempts to convey bad news [39]. In response to the death of their patients, GPs and general medical residents have reported feelings of powerlessness, guilt, sadness, loneliness, doubt and fear regarding the possibility of making a mistake [40, 41]. In addition, the difficulty of establishing a triangular relationship with the patient and the patient's family could be a source of stress with respect to the relationship with the family [42].

Another finding from the literature was the wish expressed by the families for a manifestation of support from the general practitioner after death because this attention soothes the pain [16, 33, 43].

### 4.4 Prospects for Care and Training

Our study, which focused on listening to the experiences of families, suggested that they could be involved in the process of teaching medical students. This approach may be beneficial with respect to the development of students' skills, particularly during simulated lessons [44, 45]. The development of guides and training sessions in the context of videoconferencing could help doctors enhance their skills in this context [14, 46].

## 5 Conclusion

The COVID‐19 pandemic, including the corresponding health restrictions and novel methods of remote communication, changed the ways in which families experience being informed of the death of a loved one, leading to feelings of frustration, fear, sadness and even anger. The families who participated in this research would have preferred to engage in more effective communication with caregivers, both at the time of the announcement and beforehand, which could mitigate the traumatic impact of the loss of relationships. They proposed recommendations for improving their experiences of such an announcement, highlighting the need for families to be present alongside the patient at the end of life, the use of effective telecommunication alongside the involvement of dedicated staff, and the provision of close emotional support after the announcement.

## Author Contributions

**Axelle Avazeri:** conceptualisation, investigation, writing – original draft, methodology, formal analysis, data curation. **Juliette Bonin:** conceptualisation, writing – original draft, methodology, validation, formal analysis. **Axelle Maneval:** conceptualisation, writing – review and editing, supervision. **Philippe Vorilhon:** conceptualisation, validation, methodology, writing – review and editing, supervision.

## Ethics Statement

This study did not fall within the scope of French regulations governing research involving human subjects (RIPH). A favourable ethical opinion was obtained from the Comité de protection des personnes (CPP) Sud Est VI.

## Consent

After receiving information on the conditions and objectives of the study, participants signed a written consent form.

## Conflicts of Interest

The authors declare no conflicts of interest.

## Supporting information

Supporting information.

## Data Availability

The datasets used and/or analysed during the current study are available from the corresponding author upon reasonable request.
