# Supplementary material for: Announcements of Death During the COVID‐19 Pandemic: A Qualitative Study of Family Experiences
Source: Health Expect. 2025 Mar 12;28(2):e70221. doi: 10.1111/hex.70221 (PMC11898220; doi:10.1111/hex.70221)
Supplement: Supplementary file 1 — Supporting information. [file HEX-28-e70221-s001.docx]

**Supplementary Information**

**Table of contents**

1. COnsolidated criteria for REporting Qualitative research (COREQ) checklist

2. Positionality of the Research Team

3. Final interview guide

**Supplementary Information 1. COnsolidated criteria for REporting Qualitative research (COREQ)- 32-item checklist**

| **Item No** | **Guide Questions/Description** | **Reported on Page #** |  |  |
| --- | --- | --- | --- | --- |
| **Domain 1: Research team and reflexivity** | | |  |  |
| **Personal Characteristics** | | |  |  |
| 1. Interviewer/facilitator | Which author/s conducted the interview or focus group? | Pg 5 |  |  |
| 2. Credentials | What were the researcher’s credentials? E.g., PhD, MD | Pg Supplementary information 1 |  |  |
| 3. Occupation | What was their occupation at the time of the study? | Pg Supplementary information 1 |  |  |
| 4. Gender | Was the researcher male or female? | Pg Supplementary information 1 |  |  |
| 5. Experience and training | What experience or training did the researcher have? | Pg Supplementary information 1 |  |  |
| **Relationship with participants** | | |  |  |
| 6. Relationship established | Was a relationship established prior to study commencement? | Pg 5 |  |  |
| 7. Participant knowledge of the interviewer | What did the participants know about the researcher? e.g. personal goals, reasons for doing the research? | Pg 12 |  |  |
| 8. Interviewer characteristics | What characteristics were reported about the interviewer/facilitator? e.g. Bias, assumptions, reasons and interests in the research topic | Pg 12, Supplementary information 1 |  |  |
| **Domain 2: study design** | | |  |  |
| **Theoretical framework** | | |  |  |
| 9. Methodological orientation and Theory | What methodological orientation was stated to underpin the study? e.g. grounded theory, discourse analysis, ethnography, phenomenology, content analysis | Pg 4 |  |  |
| **Participant selection** | | |  |  |
| 10. Sampling | How were participants selected? e.g., purposive, convenience, consecutive, snowball | Pg 4 |  |  |
| 11. Method of approach | How were participants approached? e.g., face-to-face, telephone, mail, email | Pg 5 |  |  |
| 12. Sample size | How many participants were in the study? | Pg 4, 6 |  |  |
| 13. Nonparticipation Setting | How many people refused to participate or dropped out? Reasons? | Pg 6, 12 |  |  |
| 14. Setting of data collection | Where was the data collected? e.g., home, clinic, workplace | Pg 5 |  |  |
| 15. Presence of nonparticipants | Was anyone else present besides the participants and researchers? | No |  |  |
| 16. Description of sample | What are the important characteristics of the sample? e.g. demographic data, date | Pg 4,5 |  |  |
| **Data collection** | | |  |  |
| 17. Interview guide | Were questions, prompts, and guides provided by the authors? Was it pilot tested? | Pg 5, Supplementary information 2 |  |  |
| 18. Repeat interviews | Were repeat interviews carried out? If yes, how many? | No |  |  |
| 19. Audio/visual recording | Did the research use audio or visual recording to collect the data? | Pg 5 |  |  |
| 20. Field notes | Were field notes made during and/or after the interview or focus group? | Pg 5, 13 |  |  |
| 21. Duration | What was the duration of the interviews or focus group? | Pg 6 |  |  |
| 22. Data saturation | Was data saturation discussed? | Pg 6 |  |  |
| 23. Transcripts returned | Were transcripts returned to participants for comment and/or correction? | Pg 6, 7, 13 |  |  |
| **Domain 3: analysis and findings** | | |  |  |
| **Data analysis** | | |  |  |
| 24. Number of data coders | How many data coders coded the data? | Pg 6 |  |  |
| 25. Description of the coding tree | Did the authors provide a description of the coding tree? | Pg 6 |  |  |
| 26. Derivation of themes | Were themes identified in advance or derived from the data? | Pg 6, 7 |  |  |
| 27. Software | What software, if applicable, was used to manage the data? | N/A |  |  |
| **Reporting** |  |  |  |  |
| 29. Quotations presented | Were participant quotations presented to illustrate the themes/findings? Was each quotation identified? e.g., participant number | Pg 7-11 |  |  |
| 30. Data and findings consistent | Was there consistency between the data presented and the findings? | Pg 7-11 |  |  |
| 31. Clarity of major themes | Were major themes clearly presented in the findings? | Pg 7-11 |  |  |
| 32. Clarity of minor themes | Is there a description of diverse cases or a discussion of minor themes? | Pg 7-11 |  |  |

Developed from: Tong A, Sainsbury P, Craig J. Consolidated criteria for reporting qualitative research (COREQ): a 32-item checklist for interviews and focus groups. International Journal for Quality in Health Care. 2007. Volume 19, Number 6: pp. 349–357

**Supplementary Information 2. Positionality of the Research Team**

Given that our identities can influence our approach, the authors wish to provide the readers with the following information about our backgrounds. During his hospital internship at the end of his 3rd cycle of medical studies, JA was confronted with difficulties in supporting families during the pandemic. This was her first experience of qualitative research; therefore, she underwent training in qualitative research. All the other researchers involved in the study were teachers in hospitals or general practices and had experience in qualitative research. JB supervises medical students in hospital placements and is particularly interested in the doctor‒patient relationship at the end of life. AM is a clinical psychologist in a palliative care unit, working with families and caregivers and conducting qualitative research at the end of life. PV is a teacher in the Department of General Medicine and conducts research on doctor‒patient relationships.

**Supplementary Information 3. Final interview guide**

**Final interview guide**

1. **Circumstances of death:**

Can you tell me about the circumstances of your loved one's death? *(Age of the deceased? Cause of death? When? Where?)*

1. **Announcement circumstances of death**

How did you find out about the death of your loved one and tell me about this announcement *(who informed you?)*

1. **Feelings**

How did you experience this announcement of the death? What emotions did you feel? *(Sadness? Anger?)*

1. **Helpful factors and difficulties**

During this announcement, what did you find helpful and difficult for you?

1. **Improvement prospects**

What do you think could have been improved during this announcement?

1. **Expectations**

What did you expect from professional caregivers in this difficult moment?

1. **Sociodemographic data of the participants’ relatives**

Gender, age, profession, place of living, relationship with the deceased person, support person or not
